# CAD/POLD2 gene expression is associated with poor overall survival and chemoresistance in bladder urothelial carcinoma

**DOI:** 10.18632/oncotarget.25701

**Published:** 2018-07-03

**Authors:** Kevin B. Givechian, Chad Garner, Hermes Garban, Shahrooz Rabizadeh, Patrick Soon-Shiong

**Affiliations:** ^1^ NantOmics LLC, Culver City, CA 90232, USA; ^2^ NantBioscience, Inc. | NantWorks, Culver City, CA 90232, USA

**Keywords:** DNA repair, chemoresistance, pyrmidine synthesis, bladder carcinoma, survival

## Abstract

Somatic mutations in DNA repair genes have been clinically associated with chemosensitivity, although few studies have interrogated the nucleotide synthesis pathways that supply DNA repair processes. Previous work suggests that bladder urothelial carcinoma is uniquely enriched for mutations in nucleotide excision repair genes, and that these mutations are associated with response to platinum-based therapy and favorable survival. Conversely, the *de novo* pyrimidine synthesis pathway has recently emerged as a putative clinical target. This anabolic process is thought to supply DNA repair processes such as nucleotide excision repair; that is, DNA repair enzymes may require a sufficient nucleotide supply available to reverse the intended genotoxic damage of systemic chemotherapy in rapidly proliferating cancer cells. Therefore, we explored the prognostic complementarity between *de novo* pyrimidine synthesis and nucleotide excision repair expression in a total of 570 bladder urothelial carcinoma patients. Ultimately, we show that the *de novo* pyrimidine synthesis gene CAD is associated with poor survival (P = 0.008) and is co-altered with the nucleotide excision repair gene POLD2. High expression of POLD2 was also associated with poor overall survival (P = 0.019) and was significantly correlated with CAD expression in pre-treatment patient tumor samples (P = 2.44e-4). Expression of each gene was associated with cisplatin-based therapy resistance, and accordingly, CAD^high^POLD2^high^ patients were associated with worse survival than CAD^high^POLD2^low^ and CAD^low^POLD2^high^ patients. Together, these biomarkers could help elucidate mechanisms of chemoresistance to further personalize therapeutic strategies in bladder urothelial carcinoma.

## INTRODUCTION

The implications of DNA repair gene alterations have recently emerged to help better stratify urothelial cancer patients by predicted response to systemic chemotherapy [1–3]. First-line systemic chemotherapy for urothelial carcinoma, as with other cancers, is used to trigger cell death in rapidly proliferating cells by forming DNA adducts that interfere with DNA replication and transcription. Accordingly, somatic gene alterations that render DNA repair enzymes defective are associated with improved response to systemic chemotherapy and survival [1, 3]. While mutations in the genes of these repair pathways have been implicated in patient prognosis and response to platinum-based chemotherapy [1, 3, 4], the complementary analysis of DNA repair and nucleotide supply remains relatively unexplored in urothelial carcinoma [5, 6].

Nucleotide production consists of many complex biochemical processes that are intertwined with feedback mechanisms to appropriately adapt to the metabolic needs of a cell. In regards to chemotherapy response, recent work has specifically highlighted the ability of cancer cells to exploit the adaptive nature of the *de novo* pyrimidine synthesis (PS) pathway for their own malignant benefit [5]. This pathway was found to be inducible by chemotherapy in triple-negative breast cancer, wherein targeting the pathway in a combination therapy rendered cancer cells sensitive to chemotherapy [5]. However, despite the malignant implication of CAD during aspartate diversion, the prevalence of DNA repair alterations during chemotherapy treatment, and the activation of *de novo* NAD+ synthesis for DNA repair during tumor progression (all of which were observed in bladder cancer) [2–8], the *de novo* PS pathway has not yet been clinically explored in bladder urothelial carcinoma (BLCA).

A recent study examining DNA repair alterations across 21 TCGA cancer cohorts, showed that BLCA was significantly associated with DNA repair alterations via the mechanism of nucleotide excision repair (NER) [8]. Defects in this repair pathway have also been found to be correlated with favorable survival and response to systemic chemotherapy [3, 8]. At the level of differential gene expression, prognostic studies of the various NER genes in BLCA are promising albeit few [4, 8]. To this end, analysis of *de novo* PS gene expression and their prognostic value in BLCA has been seemingly overlooked to date. Owing to the relatively unexplored *de novo* PS pathway in cancer, we explored the clinical relevance of *de novo* PS expression in BLCA. In the present study, we sought to implement a multifactorial prognostic analysis of *de novo* PS gene expression, while also accounting for the potentially complementary NER pathway. Lastly, we used drug-response analysis to offer putative explanations for our prognostic observations.

## RESULTS

### De novo pyrimidine synthesis genes related to OS

**Figure 1:**
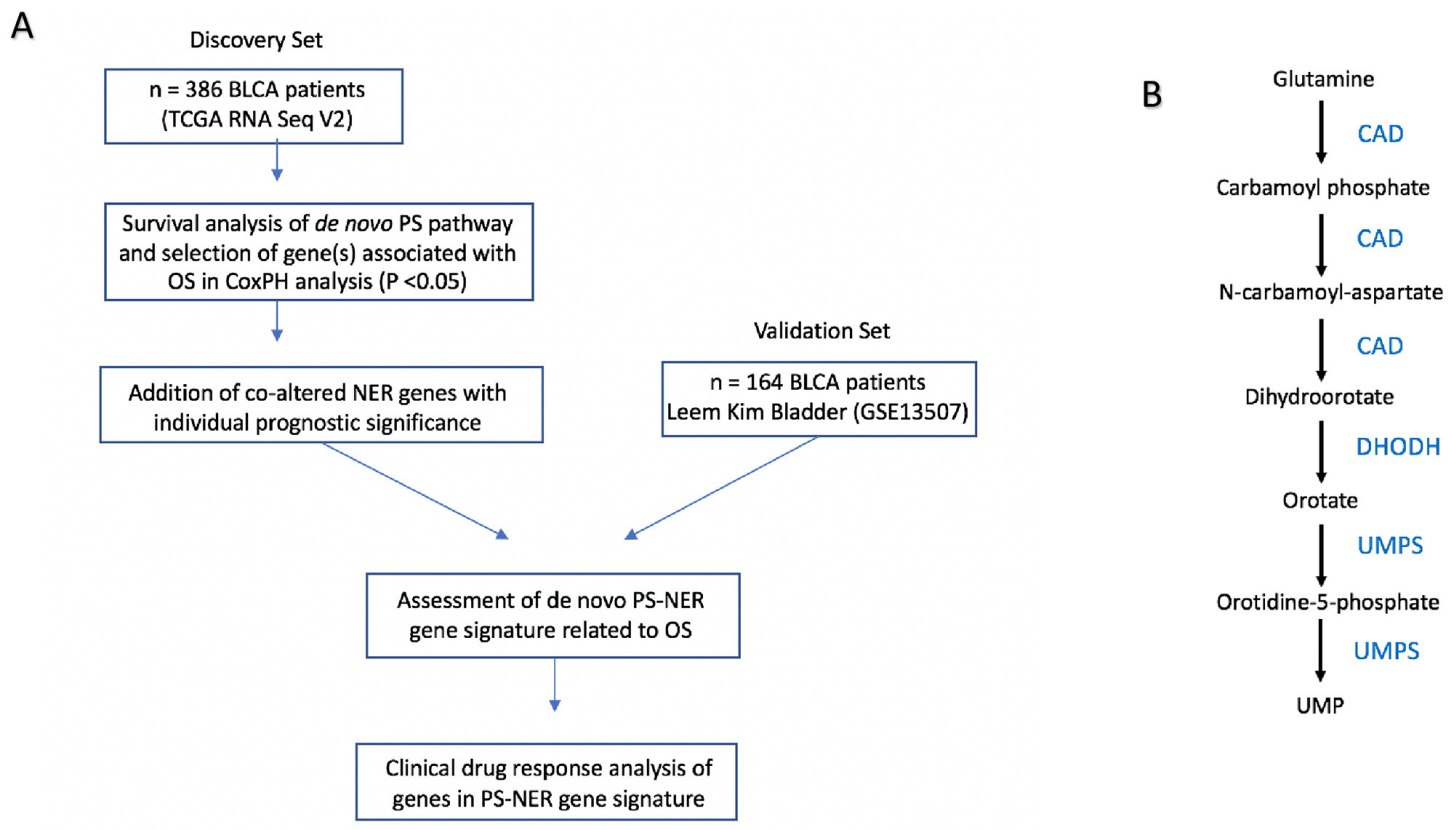
Overall workflow of the study **(A)** Workflow of research design. **(B)** Pathway for *de novo* pyrimidine synthesis.

**Figure 2:**
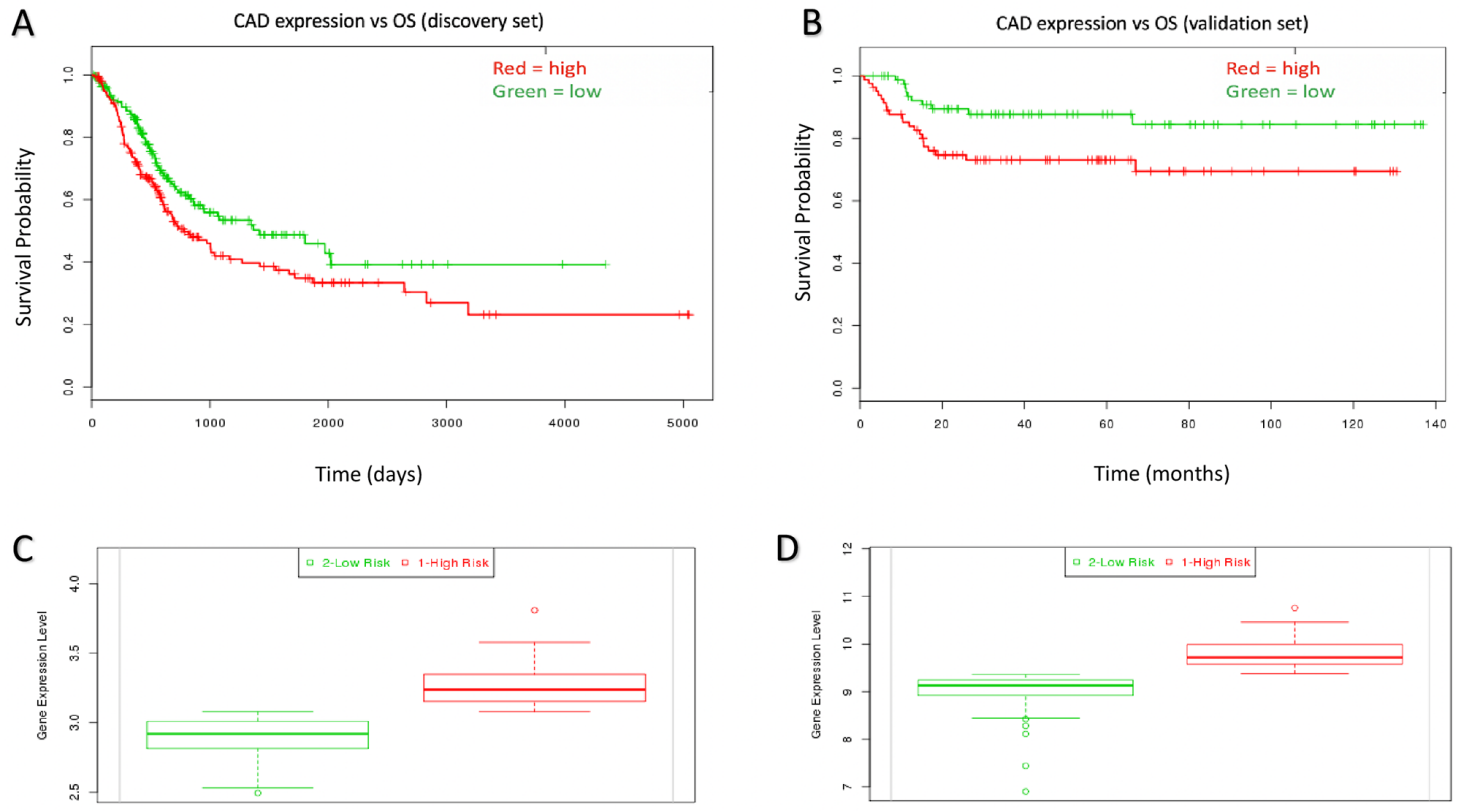
Kaplan-Meir curves for individual prognostic effect of CAD gene expression related to OS in bladder urothelial cancer patients **(A)** High expression of CAD was associated with poor prognosis (P = 0.008) in the discovery dataset. **(B)** High expression of CAD was associated with poor prognosisin the validation dataset (P = 0.017). **(C)** CAD expression relative to low/high risk group in the discovery set (P < 0.001). **(D)** CAD expression relative to low/high risk group in the validation set (P < 0.001).

The experimental workflow is shown in Figure 1A. Figure 1B shows the *de novo* pyrimidine synthesis pathway. Of the three genes in the de novo PS pathway, only CAD was associated with poor survival in the discovery set (P = 0.008; HR = 1.44, 95% CI: 1.06 – 1.95; Table 1). The prognostic significance of CAD was confirmed in the validation set (P = 0.017; HR = 2.42, 95% CI: 1.14 – 5.11; Table 1). Kaplan-Meir plots show the prognostic effect of CAD expression in the discovery and validation sets, with a median expression cutoff for high/low expression groups (Figure 2A–2B, respectively). Boxplots show differential gene expression by risk group for CAD in the discovery (P < 0.001) and validation set (P < 0.001; Figure 2C-2D, respectively).

**Table 1:** Cox proportional hazards model results for *de novo* PS gene expression

Dataset	Discovery (n = 386)				Validation (n = 164)			
	HR (95% CI)	P-value	Regression coefficient	Risk group expression	HR (95% CI)	P-value	Regression coefficient	Risk group expression
CAD^*^	1.44 (1.06-1.95)	0.008^*^	0.977	high	2.42 (1.14 – 5.11)	0.017^*^	0.715	high
*DHODH*	1.15 (0.85-1.54)	0.160	0.325	high	1.17 (0.58-2.34)	0.6631	1.25	high
*UMPS*	1.13 (0.84-1.52)	0.428	-0.346	low	1.58 (0.78-3.21)	0.1987	-0.075	low

Abbreviations: CI, confidence interval; HR, hazard ratio for risk group; ^*^ indicates significance (P < 0.05).

### Analysis of NER genes co-altered with CAD

**Figure 3:**
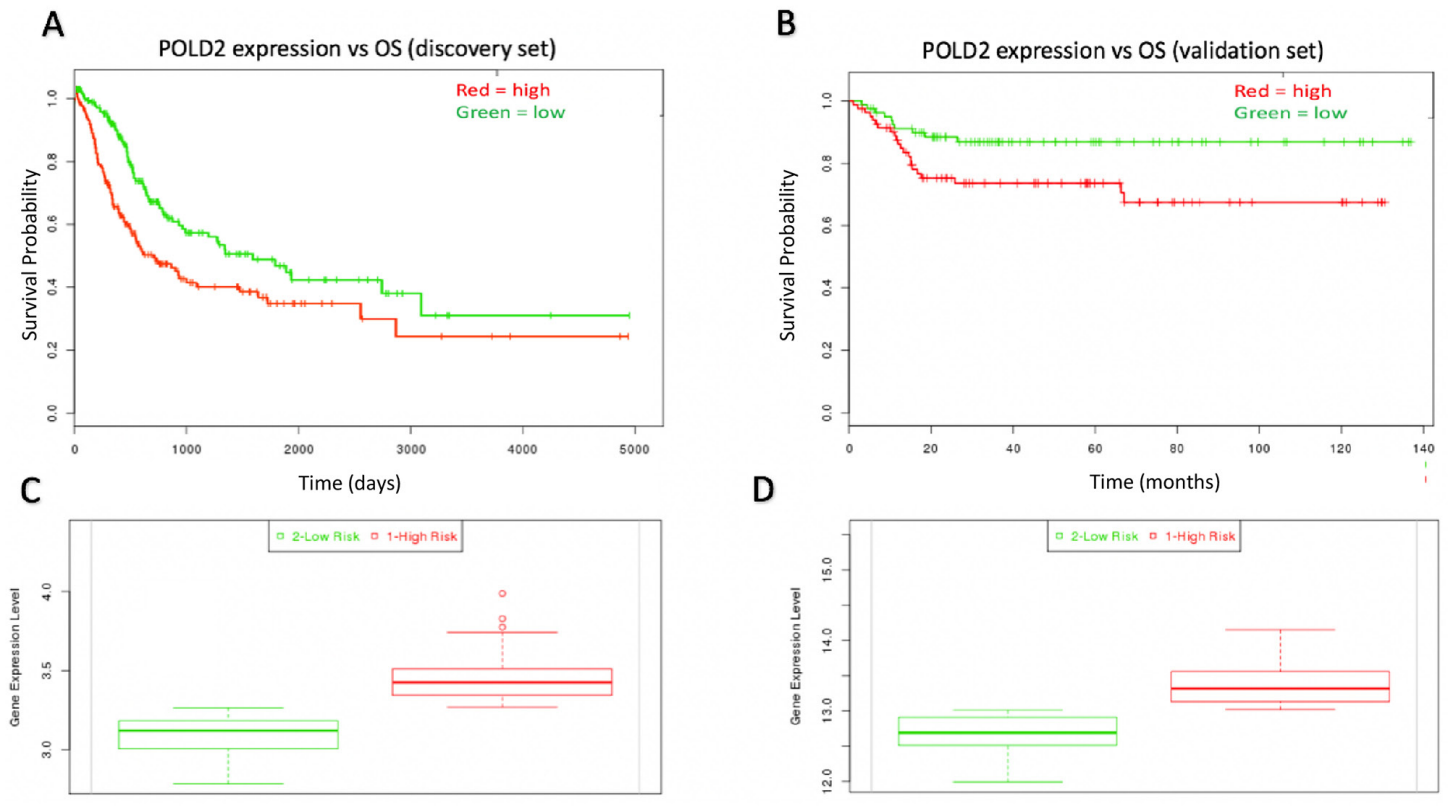
Kaplan-Meir curves for individual prognostic effect of POLD2 gene expression related to OS in bladder urothelial cancer patients **(A)** High expression of POLD2 was associated with poor prognosis (P = 0.023) in the discovery dataset. **(B)** High expression of POLD2 was associated with poor prognosisin the validation dataset (P = 0.019). **(C)** POLD2 expression relative to low/high risk group in the discovery set (P < 0.001). **(D)** POLD2 expression relative to low/high risk group in the validation set (P < 0.001).

**Figure 4:**
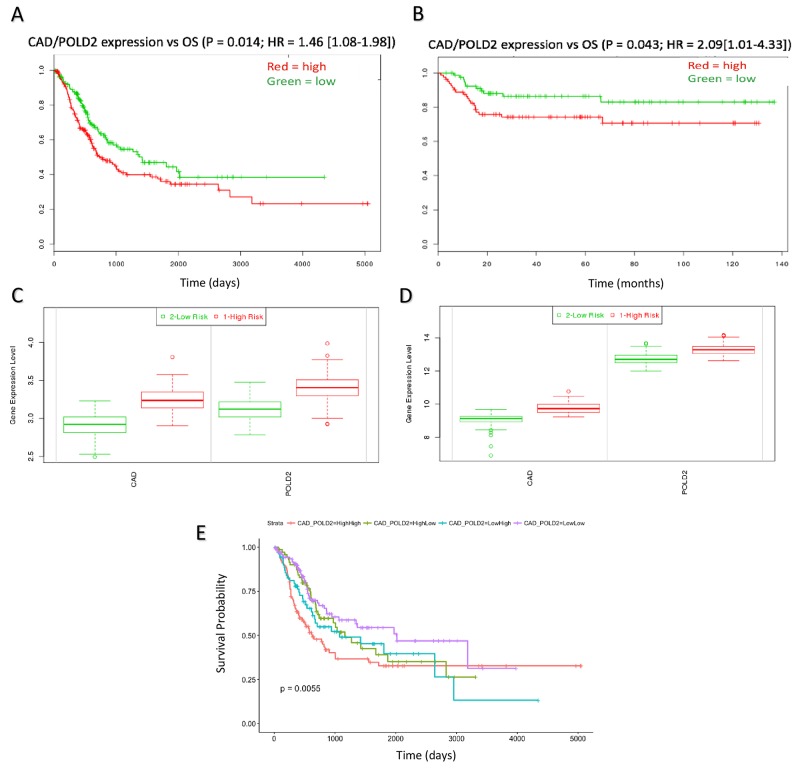
CAD/POLD2 expression analysis and independent association with drug response CAD/POLD2 expression was associated with poor prognosis in **(A)** the discovery dataset (P = 0.014) and **(B)** the validation dataset (P = 0.043). **(C)** CAD/POLD2 expressions relative to low/high risk group in the discover set (P < 0.001). **(D)** CAD/POLD2 expressions relative to low/high risk group in the validation set (P < 0.001). Multivariate model results of CAD/POLD2 expression cohorts at **(E)** full duration patient follow-up (Logrank P = 0.0019).

The Kegg Nucleotide Excision Repair gene set was used to analyze which NER genes may be associated with CAD that may also hold prognostic significance. There were 17 genes involved in NER that were significantly co-altered with CAD. This co-alteration analysis accounted for mRNA upregulation/downregulation, missense mutations, and nonsense mutations (Supplementary Table 1). An unsupervised heatmap was produced to show expression clusters of CAD and the 17 co-altered NER genes from cBioPortal [9] (Supplementary Figures 1 and 2). Each of the 17 CAD-associated NER genes was analyzed for prognostic significance in the discovery set. Of these 17 NER genes, ERCC3, ERCC5, and POLD2 each were significantly related to OS (P < 0.05; Supplementary Table 2). ERCC3 and ERCC5 had protective effects (risk group expression = low) and were not associated with response to systemic chemotherapy (data not shown), while only POLD2 expression was associated with unfavorable prognostic effect and drug resistance (risk group expression = high, P = 0.023; HR = 1.40, 95% CI: 1.04 – 1.98; Figure 3A, and Figure 4D, respectively). ERCC2 and ERCC5 were therefore excluded from further analysis. To validate the prognostic significance of POLD2 expression, OS analysis shows POLD2 expression associated with poor survival in the validation dataset (P = 0.019; HR = 2.38, 95% CI: 1.13 – 5.03; Figure 3B). The high-risk group patients in both the discovery set and validation sets possessed higher expression of POLD2 (P < 0.001; Figure 3C-3D).

### Multifactorial analysis of CAD/POLD2 expression related to OS

When combined, CAD and POLD2 gene expression was associated with poor OS in both the discovery and validation datasets (P = 0.014; HR = 1.46, 95% CI: 1.08 – 1.98 and P = 0.043; HR = 2.09, 95% CI: 1.01 – 4.43, respectively; Figure 4A-4B). The high-risk group patients (PI > median) in both the discovery set and validation sets possessed higher expression of CAD/POLD2 (P < 0.001; Figure 4C-4D). We also fit a multivariate model and showed that patients that possessed both high CAD and high POLD2 expression together exhibited the worst overall survival (Logrank P = 0.0019; Figure 4E, Supplementary Table 3).

### CAD and POLD2 association with patient drug response

**Figure 5:**
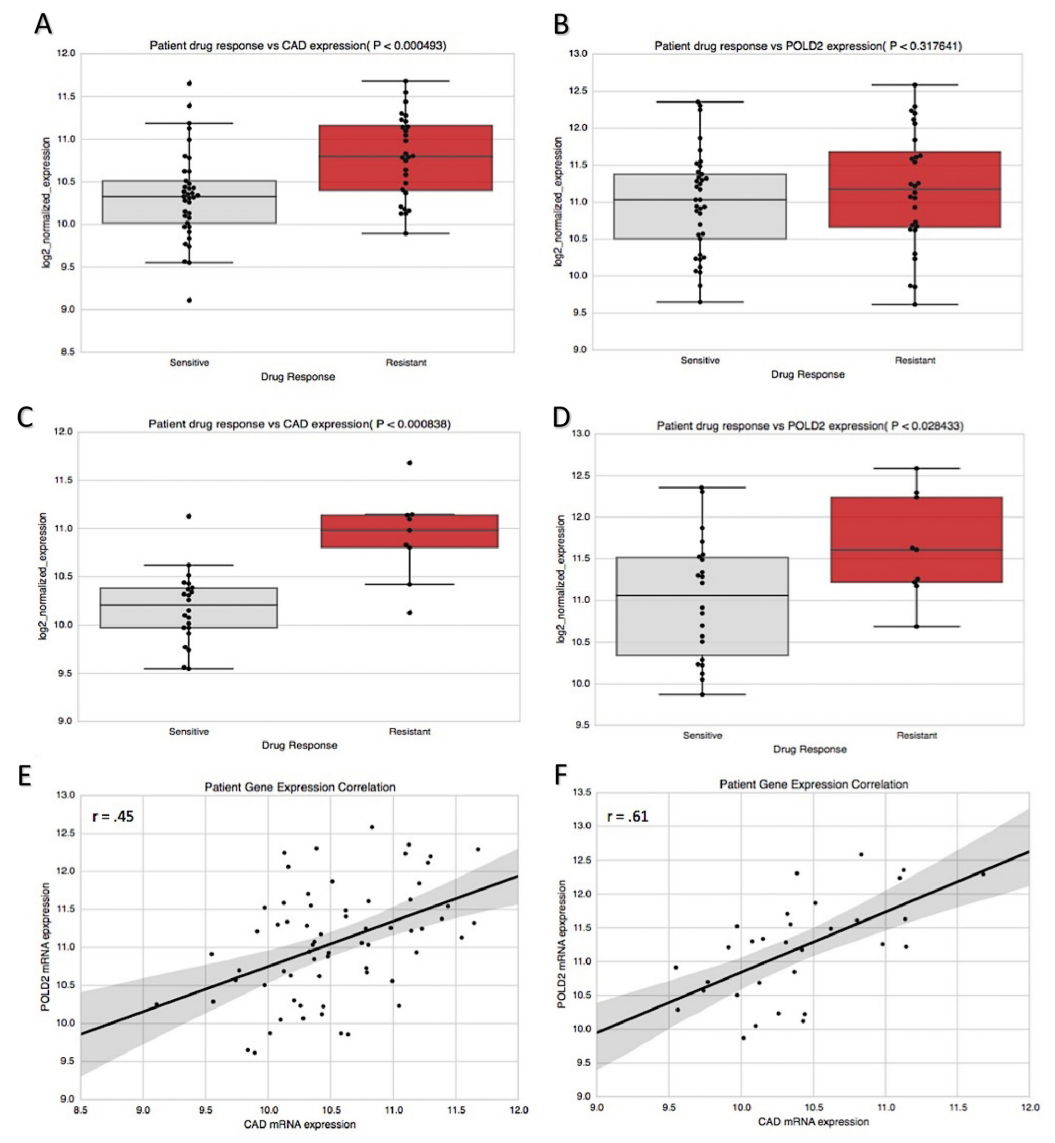
CAD and POLD2 association with chemotherapy response in bladder cancer patients **(A)** Patients resistant to systemic chemotherapy possessed higher CAD expression (P = 4.93e-4), **(B)** but this was not true for POLD2 (P = 0.312). **(C)** however, when analysis was restricted to cisplatin-based therapy regimen, CAD was associated with drug resistance (P = 8.38e-4) and **(D)** this was also true for POLD2 (P = 0.028). Patient expression of CAD and POLD2 was significantly correlated in **(E)** tumor samples for patients administered systemic chemotherapy (r = 0.45, P = 1.89e-4). and **(F)** this correlation was strengthened when restricted to cisplatin-based therapy (r = 0.61, P = 2.44e-4).

When CAD and POLD2 were examined for their association with drug response data in BLCA [10], CAD expression associated with resistance to systemic chemotherapy (P = 4.93e-4; Figure 5A), but this did not hold true for POLD2 (P = 0.318; Figure 5B). Interestingly, however, POLD2 has been implicated in cellular resistance specifically to cisplatin, due to its ability to dramatically increase the efficiency and processivity of DNA synthesis via interaction with Pol ζ4 in order to bypass 1,2-intrastrand d(GpG)-cisplatin cross-links [11, 12]. In light of this, we examined whether the unfavorable prognostic effects of POLD2 may instead be specifically through resistance to cisplatin-based therapy, which is a standard first-line therapy in BLCA. In patients treated with cisplatin-based therapy, CAD and POLD2 were both significantly associated with cisplatin-based therapy resistance (P = 8.38e-4 and P= 0.028, respectively; Figure 5C-5D), suggesting that, unlike for CAD, the chemoresistant effects of POLD2 may be specific to cisplatin-based therapy. To determine the extent to which CAD and POLD2 patient expressions were correlated in samples of our drug response analysis, we examined Pearson correlation coefficients. CAD and POLD2 were significantly correlated in BLCA tumor samples; however, when restricted to patients administered cisplatin-based therapy, patient expressions became more tightly correlated (r = 0.45, P < 0.001 vs r = 0.61, P < 0.001, respectively; Figure 5E-5F).

## DISCUSSION

In the current study, we analyzed the prognostic relationship of PS and NER gene products in BLCA, and we have shown that CAD/POLD2 gene expression is associated with poor OS, perhaps in part due to chemoresistance. Upon observing the implication of pyrimidine synthesis genes in BLCA OS, CAD became of critical interest. The proceeding two genes of the *de novo* PS pathway, namely DHODH and UMPS, were not associated with OS perhaps because they independently catalyze fewer steps of the pathway, while CAD catalyzes the first three steps of *de novo* PS. Intriguingly, CAD is also associated with unfavorable survival in liver cancer and renal cancer [13], and it catalyzes the rate-limiting step of the *de novo* PS pathway [14], suggesting it may be expressed at higher levels than DHODH and UMPS in *de novo* PS to ameliorate chemotherapy induced genotoxic damage. Our prognostic observations of CAD are also in line with its amplification as a marker of genomic instability in tumorigenic liver cells, its association with mutant TP53 status, and its implication in cancer cell viability in BLCA and TNBC [5, 6, 15, 16]. We therefore believe the objective catalytic involvement of CAD in pyrimidine production may in part be to supply NER enzymes the re-building blocks necessary to repair genotoxic damage from systemic chemotherapy, as has been demonstrated in the context of DNA replication [17]. Providing sufficient nucleotides for NER may in turn mitigate the intended pro-apoptotic effects of chemotherapeutic compounds, offering a biological explanation for our prognostic observations.

When examining co-altered NER genes for their prognostic relevance, ERCC3 and ERCC5 were observed to possess protective effects in BLCA. While this may seemingly challenge previous evidence that higher excision repair gene expression is associated with worse OS [2, 4, 18], these studies examined ERCC1 and ERCC2 as opposed to the complementation groups (3 and 5) revealed by our CAD co-alteration analysis, suggesting a context-dependent clinical effect for varying excision repair complementation groups. Moreover, the prognostic signal that manifested for ERCC3 and ERCC5 was not corroborated by drug response analysis, unlike that which we observed for POLD2. Of note, we found that ERCC2 alterations (which are recurrently found in bladder cancer) co-occur significantly with POLD2 expression (P = 0.010; data not shown), but not with CAD expression, suggesting POLD2 as putative gene of interest in future studies examining ERCC2.

We observed POLD2 to be associated with poor survival in BLCA and cisplatin-based therapy resistance. POLD2 is a subunit of the DNA polymerase delta exonuclease complex and is known to play a crucial role in NER [11]. Additionally, POLD2 has been implicated in ovarian carcinogenesis as well as poor glioma patient prognosis [19–24]. This catalytic subunit has also been associated with poor survival in serous carcinoma, as well as 1,2-intrastrand d(GpG)-cisplatin cross-link bypass via improved Pol ζ efficiency and cooperativity [12, 24]. Therefore, our observations offer a plausible mechanism by which pro-apoptotic cisplatin-based therapy DNA damage is ameliorated by higher expression of POLD2 and CAD, which help bypass cisplatin-induced DNA adducts and maintain a sufficient pyrimidine pool for repair, respectively. Of note, multivariate analysis revealed that POLD2 expression (which is moderately correlated with CAD in the TCGA BLCA Provisional dataset; r = 0.37, data not shown [9]), was associated with the worse overall survival. This may therefore suggest that the detrimental effect of high CAD/POLD2 co-expression is pronounced early in the course of the disease when patients are generally more aggressively treated with systemic chemotherapy regimens such as cisplatin-based therapy [11]. Therefore, an interesting hypothesis to pose for future studies is the possibility that the unfavorable prognostic effect of CAD/POLD2 co-expression is driven by the ability to suppress the pro-apoptotic effects of chemotherapy.

It is also worth considering these results in light of neoantigen burden and immunogenicity, as recent work has shown that inactivating DNA repair processes can increase the amount of neoantigens and impair tumor growth in colorectal cancer, breast adenocarcinoma, and pancreatic ductal carcinoma [25]. Furthermore, we have previously reported a pan-cancer transcriptomic approach that distinguished a relatively non-immunogenic cluster of patient tumor samples with elevated expression of nucleotide metabolism, mismatch repair, and DNA damage response pathways [26]. This group of patients, which included but was not limited to bladder urothelial carcinoma, was also shown to have worse overall survival compared to patients without the same elevated pathways. While examining mechanisms of neoantigen generation is out of the scope of the current study, it suggests an interesting link between DNA repair, pyrimidine synthesis, and immunosurveilance for future exploration.

There are a several limitations to our study due to lack of available clinical data and consequential small sample sizes, which is an issue that has also been previously encountered and addressed [27–29]. First, the lack of clinical data prevented us from examining relationship between CAD/POLD2 and tumor stage features, lymph node status, as well as progression- and relapse-free survival; the small sample sizes also prevented our ability to adequately conduct simultaneous multivariate analysis for CAD and POLD2. Second, because our analysis was inherently retrospective, distinguishing the prognostic value versus predictive power of CAD/POLD2 in cisplatin-based therapy resistance would necessitate a prospective randomized trial, with a cisplatin-free arm and appropriate gene panel for differential expression analysis. In addition, the 17 co-altered NER genes are correlated and so we did not apply a multiple test correction to their prognostic results as they are unlikely to represent independent tests. Third, tumor samples analyzed for drug response were solely pre-treatment samples, so determining whether systemic chemotherapy may be inducing CAD/POLD2 for adduct bypass is beyond the scope of our study. Nevertheless, our results offer prognostic insight in BLCA and may also encourage the required efforts of clinical annotation in future genomic studies. In light of the clinical data limitations, we specifically pursued a large validation dataset with gene expression profiled by a different platform (e.g., RNA-seq V2 vs affymetrix microarrays) to mitigate potential false positives by obtaining our results across different platforms [27].

Our study conclusively demonstrates a systematic *de novo* PS-based approach that considers the complementary biology between pyrimidine production and NER, which is often significantly altered in BLCA. From the three *de novo* PS genes and those of the NER pathway, our analysis identified two genes (CAD, POLD2) that were both independent prognostic factors. Analyzing combined expression of these genes in a multifactorial model revealed association with poor clinical outcome and chemoresistance. These results encourage prospective clinical validation and reveal the utility of accounting for *de novo* pyrimidine synthesis in the clinical context of chemosensitivity.

## MATERIALS AND METHODS

### Discovery and validation sets

In the discovery set, we examined 386 patient primary tumor samples with available clinical survival data and RNA-seq V2 expression data in the TCGA bladder urothelial carcinoma (BLCA) 2016 dataset via SurvExpress [28] (clinical characteristics available at http://www.cbioportal.org/data_sets.jsp). These patients were evaluated for overall survival (OS) relative to primary tumor gene expression. In the validation set, we examined 164 primary patient bladder cancer samples that were expression profiled by array (clinical characteristics available via GEO accession GSE13507).

### Pyrimidine synthesis and NER gene selection

Pyrimidine synthesis genes selected for initial analysis were the genes of the *de novo* pyrimidine synthesis pathway. The NER gene set used for preliminary analysis was the Kegg Nucleotide Excision Repair pathway (hsa03420), which contained 44 genes. These genes were evaluated for their degree of co-alteration with CAD through the cBioPortal mutual exclusivity and co-occurrence module, using the TCGA Provisional dataset (n=408) [9]. P-values were derived from Fisher’s exact test. The log odds ratio quantifies how strongly the presence or absence of alterations of two genes are associated in the tumor samples (log odds ratio > 0 = association towards co-occurrence; log odds ratio <= 0 association towards mutual exclusivity). Subsequently, Kegg NER genes selected for prognostic analysis in the discovery and validation set were restricted to those significantly co-altered with CAD (a total 17 genes were tested for prognostic association). Multiple-testing correction was not performed because the 17 NER genes examined are correlated in expression (as shown by the supplemental heatmap), likely because of their activity in the same pathway, so they do not represent independent tests. The OncoPrint visualization was generated in cBioPortal [9], and the unsupervised expression heatmap and corresponding denogram were generated in R using the ComplexHeatmap library.

### Independent gene survival analysis

Gene expression was evaluated to determine those strongly associated with OS (P < 0.05) using a CoxPH model in R via SurvExpress to determine hazard ratio relative to the risk group [9]. The data of each set was the original (quantile-normalized) data, and for the validation microarray set, all probe sets were averaged per sample (e.g., if multiple probe sets existed for a gene). Samples were ordered according to prognostic index (PI) each patient and separated into risk group cohorts by a median split. The formula used to generate the prognostic index is below:

PI = *βx*

where *β* can be interpreted as a risk/linear regression coefficient for *x*, which is the expression value for a gene of interest in a given tumor sample. *β* for each gene was obtained from the Cox fitting. OS was shown by Kaplan-Meir (KM) plots. KM Plots were generated with cohorts segregated by risk groups by the PI median relative to high versus low gene expression, and survival curves were generated and compared using the log-rank test.

### CAD/POLD2 multifactorial survival analysis

For discovery and validation of CAD/POLD2 prognostic significance, regression coefficients were individually obtained from the discovery data set models. These regression coefficients served as the weights in the final CoxPH validation model in which risk group cohorts were separated at the median PI for the cutoff as shown by:

PI_*CAD/POLD2*_ = *β*_*CAD*_*x*_*CAD*_ + *β*_*POLD2*_*x*_*POLD2*_

where *β*_*CAD*_ = 0.977 and *β*_*POLD2*_ = 0.715. Patient OS relative to patient expression of CAD/POLD2 was shown by the Kaplan-Meir (KM) method. KM Plots were generated with cohorts segregated by risk groups by the PI median relative to high versus low gene expression of CAD and POLD2 in the final linear model [30], and survival curves were generated and compared using the KM method and the log-rank test. The survival and survminer packages were used to conduct multivariate Cox regression analysis of the TCGA data set in R with median expression cutoffs.

### Clinical drug response analysis

Curated records of drug treatments and outcomes generated from TCGA clinical data [10] were used to analyze the differential gene expressions of BLCA patients who were sensitive or resistant to systemic chemotherapy. There was a total of 65 BLCA patients with clinical drug-response annotation to systemic chemotherapy and corresponding pre-treatment log2-normalized RNA-seq V2 expression data (responders: n = 37, non-responders: n = 28). There was a total of 31 BLCA patients with clinical response labels to cisplatin-based therapy and corresponding pre-treatment log2-normalized mRNA-seq expression data (responders: n = 22, non-responders: n = 9). Two-tailed *t*-tests were used to determine differential expression significance, and Pearson r values were calculated for log2-normalized patient gene expression correlation analysis.

## SUPPLEMENTARY MATERIALS FIGURES AND TABLES



## Abbreviations

BLCA: bladder urothelial carcinoma
OS: overall survival
PS: pyrimidine synthesis
CAD: carbamoyl-phosphate synthetase 2, aspartate transcarbamylase, and dihydroorotase
DHODH: dihydroorotate dehydrogenase
UMPS: uridine monophosphate synthase
POLD2: DNA polymerase delta subunit 2
NER: nucleotide excision repair
