# Supplementary material for: CAD/POLD2 gene expression is associated with poor overall survival and chemoresistance in bladder urothelial carcinoma
Source: Oncotarget. 2018 Jul 3;9(51):29743–52. doi: 10.18632/oncotarget.25701 (PMC6049856; doi:10.18632/oncotarget.25701)
Supplement: Supplementary file 1 [file oncotarget-09-29743-s001.pdf]

CAD/POLD2 gene expression is associated with poor overall survival and chemoresistance in bladder urothelial carcinoma

SUPPLEMENTARY MATERIALS

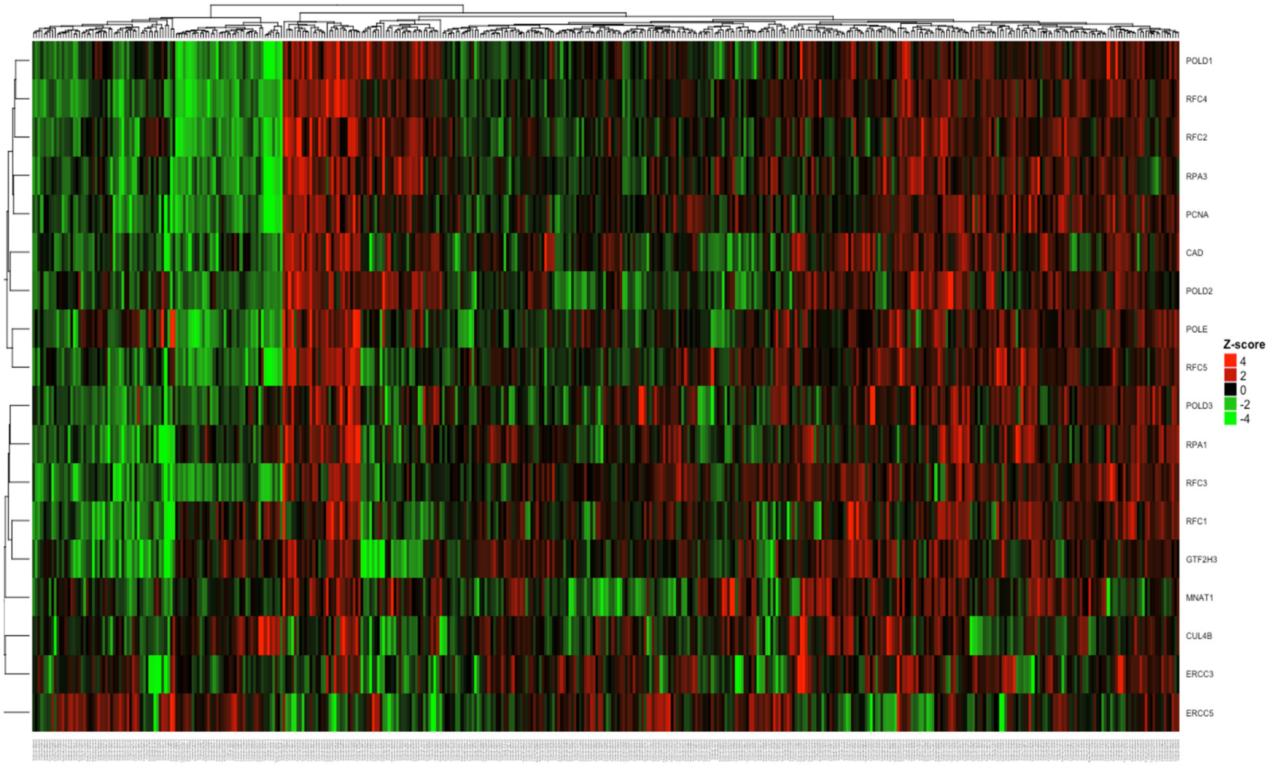

Supplementary Figure 1: Heatmap clustering of CAD and CAD-associated nucleotide excision repair genes in TCGA BLCA patient tumor samples. Hierarchal clustering of the 17 co-alteration/mutual-exclusive NER genes and CAD to visualize clusters by patient expression z-scores. Columns correspond to TCGA patients and rows correspond to genes.

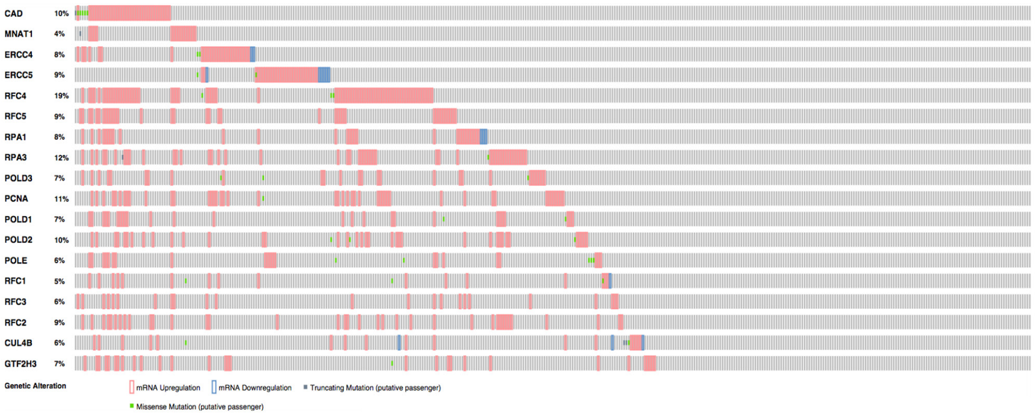

Supplementary Figure 2: OncoPrint visualization of CAD and CAD-associated nucleotide excision repair genes in TCGA BLCA patient tumor samples. OncoPrint results from cBioPortal for CAD and 17 co-altered/mutually-exclusive NER genes in TCGA samples.

Supplementary Table 1: CAD co-alteration analysis with the nucleotide excision repair pathway

Gene A	Gene B	p-Value	Log Odds Ratio	Association
CAD	MNAT1	0.009172906	1.499132138	Tendency towards co-occurrence
CAD	ERCC3	0.025363905	0.852173175	Tendency towards co-occurrence
CAD	ERCC5	0.024046249	-1.834319084	Tendency towards mutual exclusivity
CAD	RFC4	2.22E-06	1.542470638	Tendency towards co-occurrence
CAD	RFC5	1.05E-05	1.746639034	Tendency towards co-occurrence
CAD	RPA1	0.033659832	0.882500409	Tendency towards co-occurrence
CAD	RPA3	0.008758494	0.965561781	Tendency towards co-occurrence
CAD	POLD3	0.014035124	1.073294481	Tendency towards co-occurrence
CAD	PCNA	0.004450951	1.114698426	Tendency towards co-occurrence
CAD	POLD1	2.79E-05	1.774638428	Tendency towards co-occurrence
CAD	POLD2	0.019814681	0.944634261	Tendency towards co-occurrence
CAD	POLE	0.016617056	1.109637759	Tendency towards co-occurrence
CAD	RFC1	0.009096674	1.355262252	Tendency towards co-occurrence
CAD	RFC3	0.006386313	1.163269511	Tendency towards co-occurrence
CAD	RFC2	0.000535725	1.438938095	Tendency towards co-occurrence
CAD	CUL4B	0.023967257	1.107345969	Tendency towards co-occurrence
CAD	GTF2H3	7.79E-05	1.718898121	Tendency towards co-occurrence

cBioPortal co-alteration/mutual-exclusivity results for CAD and the genes in the Kegg NER pathway. Analysis accounted for mRNA upregulation/downregulation, missense mutations, and nonsense mutations in TCGA samples. 17 NER genes were significantly co-altered with CAD.

Supplementary Table 2: Prognostic results of the CAD-associated nucleotide excision repair genes

Gene	Discovery Set HR (95% CI)	P-value	Regression coefficient
MNAT1	1.27 (0.94-1.71)	0.120	0.43
*ERCC3**	1.45 (1.07-1.96)	0.015*	-0.437
*ERCC5**	1.68 (1.24-2.27)	0.0008*	-1.251
RFC4	0.97 (0.72-1.31)	0.856	0.01
RFC5	1.15 (0.85-1.55)	0.356	-0.217
RPA1	1.32 (0.98-1.78)	0.072	0.71
RPA3	0.88 (0.65-1.19)	0.392	-0.05
POLD3	1.03 (0.77-1.39)	0.828	0.025
PCNA	1.29 (0.96-1.74)	0.092	-0.257
POLD1	1.12 (0.83-1.52)	0.440	-0.306
*POLD2**	1.4 (1.04-1.89)	0.023*	0.715
POLE	1.12 (0.83-1.5)	0.474	-0.315
RFC1	1.03 (0.76-1.39)	0.859	0.097
RFC3	1.05 (0.78-1.42)	0.732	0.122
RFC2	0.94 (0.7-1.27)	0.694	0.234
CUL4B	1.08 (0.8-1.46)	0.606	0.263
GTF2H3	1.22 (0.91-1.65)	0.190	0.175

Abbreviations: CI, confidence interval; HR, hazard ratio for risk group; * indicates significance (P < 0.05).
Cox model results for each of the CAD-associated NER genes in the Kegg NER pathway. ERCC3, ERCC5, and POLD2 each held prognostic significance (P = 0.015, P = 0.008, P = 0.023, respectively).

Supplementary Table 3: Multivariate prognostic analysis results of CAD and POLD2 expression

Gene	Discovery Set HR (95% CI)	P-value	Regression coefficient
POLD2	1.52 (1.12-2.08)	0.007*	0.42
CAD	1.28 (0.95-1.75)	0.106	0.25

Multivariate model results for CAD/POLD2 gene expression in TCGA dataset.
